# Downregulation of Kinesin Spindle Protein Inhibits Proliferation, Induces Apoptosis and Increases Chemosensitivity in Hepatocellular Carcinoma Cells

**DOI:** 10.6091/ibj.1386.2014

**Published:** 2015-01

**Authors:** Chinh Chung Doan, Ngoc Trung Doan, Quang Huy Nguyen, Minh Hoa Nguyen, Minh Si Do, Van Dong Le

**Affiliations:** 1 *Faculty of Biology, University of Science, Vietnam National University, 227 Nguyen Van Cu Street, Ward 4, District 5, Ho Chi Minh City, Vietnam; *; 2 *Dept. of Immunology, Vietnam Military Medical University, 160 Phung Hung Street, Ha Dong District, Ha Noi City, Vietnam*

**Keywords:** Apoptosis, Chemosensitivity, Doxorubicin, Hepatocellular carcinoma (HCC) cells, Kinesin spindle protein (KSP)

## Abstract

**Background**: Kinesin spindle protein (KSP) plays a critical role in mitosis. Inhibition of KSP function leads to cell cycle arrest at mitosis and ultimately to cell death. The aim of this study was to suppress KSP expression by specific small-interfering RNA (siRNA) in Hep3B cells and evaluate its anti-tumor activity. **Methods**: Three siRNA targeting KSP (KSP-siRNA #1-3) and one mismatched-siRNA (Cont-siRNA) were transfected into cells. Subsequently, KSP mRNA and protein levels, cell proliferation, and apoptosis were examined in both Hep3B cells and THLE-3 cells. In addition, the chemosensitivity of KSP-siRNA-treated Hep3B cells with doxorubicin was also investigated using cell proliferation and clonogenic survival assays. **Results**: The expression of endogenous KSP at both mRNA and protein levels in Hep3B cells was higher than in THLE-3 cells. In Hep3B cells, KSP-siRNA #2 showed a further downregulation of KSP as compared to KSP-siRNA #1 or KSP-siRNA #3. It also exhibited greater suppression of cell proliferation and induction of apoptosis than KSP-siRNA #1 or KSP-siRNA #3; this could be explained by the significant downregulation of cyclin D1, Bcl-2, and survivin. In contrast, KSP-siRNAs had no or lower effects on KSP expression, cell proliferation and apoptosis in THLE-3 cells. We also noticed that KSP-siRNA transfection could increase chemosensitivity to doxorubicin in Hep3B cells, even at low doses compared to control. **Conclusion**: Reducing the expression level of KSP, combined with drug treatment, yields promising results for eradicating hepatocellular carcinoma (HCC) cells *in vitro*. This study opens a new direction for liver cancer treatment.

## INTRODUCTION

Hepatocellular carcinoma (HCC) is the fifth common cancer in the world. Due to the lack of an early clinical diagnosis method and obvious symptoms, HCC tumor is regularly detected at advanced stages. Although surgical operation on tumor removal is generally helpful for HCC patients, clinical mortality of HCC is relatively high because therapeutic options of HCC are rather limited [1]. Besides, traditional chemotherapies using anti-cancer drugs or radiotherapy are inefficacious for HCC patients. In many cases, this combined therapy still leaves some problems such as metastatic lesions and subsequent cancer recurrence [2]. Recently, following the development of modern molecular biology, in-depth researches have been conducted in developing new strategies of HCC treatment at genetic level. In particular, RNA interference (RNAi) may represent a powerful strategy to interfere with key molecular pathways involved in cancer and has established a new area of clinical therapy for HCC [3].

RNAi is a process in which activation of an intracellular pathway modulated by small-interfering RNA (siRNA) composed of 21-23 nucleotides (nt) leads to degradation of a specific, targeted mRNA [4]. The selective and robust effect of RNAi on gene expression makes it a valuable research tool both in cell culture and in living organisms because synthetic siRNA introduced into cells can induce suppression of specific genes of interest [5]. Another unique advantage of RNAi is that non-druggable protein targets can be efficiently knocked-down and possibly achieve therapeutic effects [6]. Therefore, siRNA-induced RNAi presents an effective and a simple method to silence a wide range of cancer-associated genes. Moreover, a number of siRNA have been established that are capable of silencing some different types of human HCC gene targets, such as cyclin E, *vascular endothelial growth factor*, COP9 signalosome subunit 5, c-Myc [7-10] and so on.

It has been known that human cancer is a gene-related disease involving abnormal cell growth. As a new member of the kinesin superfamily of microtubule -based motors, the kinesin Eg5, also called kinesin spindle protein (KSP) or KIF11 is a molecular motor that participates in mitosis, by separating the microtubules that are attached to two centrosomes, thus contributing to the bipolar arrangement of the spindle [11]. Failure to establish a bipolar spindle results in a mitotic arrest, after which cells may experience a variety of fates, including abnormal exit from mitosis, resumption of the cell cycle, and apoptosis [6]. The overexpression of KSP as a transgene may cause genomic instability and tumor formation in mice [12, 13]. Furthermore, in contrast to microtubules which are also presented in post-mitotic cells, KSP is exclusively expressed in mitotic cells, which make it an ideal target for anti-mitotics [14]. Inhibition of KSP activity leads to cell cycle arrest of mitotic cells in prometaphase with the formation of monoastral microtubule arrays and eventually to cell death [15, 16]. Therefore, several KSP inhibitors have been studied in clinical trials and provided new opportunities for development of novel anti-cancer therapeutics alternative from the available microtubule targeting drugs [17-19]. 

In this study, siRNA targeting KSP was used to reduce KSP expression in Hep3B cells and to investigate the chemosensitivity of the KSP down-regulated cells to doxorubicin. Our evidence indicates KSP-silencing efficiency and the impact of KSP downregulation combined with chemotherapy on the growth of Hep3B cells* in vitro*.

## MATERIALS AND METHODS

***Cell culture. ***Cancerous cell line Hep3B (HCC cells, HB-8064) and non-cancerous cell line THLE-3 (normal human liver cells, CRL-11233) were provided from the American Type Culture Collection (Rockville, MD, USA). Hep3B cells were thawed and cultured in DMEM, supplemented with 10% FBS, 2 mM L-glutamine, 0.1 mM MEM non-essential amino acids, 1.5 g/l sodium bicarbonate, and 0.5% antibiotic-mycotic (all were bought from Sigma-Aldrich, St. Louis, MO, USA). THLE-3 cells were thawed and cultured in bronchial epithelial cell growth medium medium (Lonza, Walkersville, MD, USA), supplemented with 10% FBS, 5 ng/ml *epidermal growth factor*, 5 ng/ml *hepatocyte growth factor*, 70 ng/ml phosphoethanolamine, and 0.5% antibiotic-mycotic (all were bought from Sigma-Aldrich, St. Louis, MO, USA). The cells were maintained in a humidified atmosphere of 5% CO_2_ at 37^o^C.

***Transient transfection of siRNA. ***The sequences of the siRNA targeting KSP (KSP-siRNA#1-3) and mismatched siRNA (Cont-siRNA) are shown in Table 1. All siRNA were synthesized by Bioneer Co., Ltd (Daejeon, Republic of Korea). Each siRNA was resuspended in nuclease-free water, and the stock solutions were stored at 4^o^C until use. The KSP-siRNA#1-3 or Cont-siRNA was transiently transfected in both Hep3B cells and THLE-3 cells with a Lipofectamine RNAiMAX Transfection Reagent kit (Invitrogen Inc., Carlsbad, CA, USA) by reverse transfection protocol. Briefly, for each well of 24-well plate (Corning Inc., NY, USA), 3 µl of siRNA duplex (20 µM) was mixed with 1 µl transfection reagent and 100 µl Opti-MEM medium supplied with hypo-xanthine, thymidine, sodium pyruvate, L-glutamine, trace elements, and growth factors. Then, the siRNA-transfection reagent complex was incubated with 500 µl of diluted cells (5 × 10^4^ cells/well) at 37°C for 24-72 h, 5% CO_2_. The cells without siRNA transfection were used as control. The siRNA-treated cells and control cells were harvested for transfection efficiency analysis during time intervals of 24-72 h.

**Table 1:** Sequences of the siRNA

**Name**	**Sequences (5’–3’)**
KSP-siRNA #1	Sense: CUGAAGACCUGAAGACAAUdTdT Antisense: AUUGUCUUCAGGUCUUCAGdTdT
KSP-siRNA #2	Sense: UCGAGAAUCUAAACUAACUdTdT Antisense: AGUUAGUUUAGAUUCUCGAdTdT
KSP-siRNA #3	Sense: CUGGAUCGUAAGAAGGCAGdTdT Antisense: CUGCCUUCUUACGAUCCAGdTdT
Cont-siRNA	Sense: GCGGAGAGGCUUAGGUGUAdTdT Antisense: UACACCUAAGCCUCUCCGCdTdT

**Table 2:** Sequences of the primers for RT-PCR and real-time qRT-PCR

**Name**	**Sequences (5’–3’)**	**Product size (bp)**
KSP	Forward: CTGAACAGTGGGTATCTTCCTTA Reverse: GATGGCTCTTGACTTAGAGGTTC	480
Cyclin D1	Forward: GCCCGAGGAGCTGCTGCAAA Reverse: CCTGGCGCAGGCTTGACTCC	358
Bcl-2	Forward: CGGTGCCACCTGTGGTCCAC Reverse: TCCCCCAGTTCACCCCGTCC	174
Survivin	Forward: GGACCGCCTAAGAGGGCGTGC Reverse: AATGTAGAGATGCGGTGGTCCTT	145
β-actin	Forward: ACACTGTGCCCATCTAGGAGG Reverse: AGGGGCCGGACTCGTCATACT	680

***Reverse transcription PCR (RT-PCR). ***Total RNA was extracted using RNeasy Mini Kit (Qiagen, Valencia, CA). The concentration of RNA was measured using a BioPhotometer (Eppendorf, Hamburg, Germany). One-step RT-PCR was performed from total RNA using Access Quick RT-PCR kit (Promega, Madison, WI, USA) under the following conditions: initial reverse transcription at 45°C for 45 min and 95°C for 2 min, followed by 35 cycles of denaturing at 94°C for 45 s, annealing at 55-58°C for 30 s, and extension at 72°C for 45 s. After completion of the last cycle, all samples were incubated at 72^o^C for 10 min. The sequences of primers are shown in Table 2. PCR products were analyzed by electrophoresis with 2% agarose gel, visualized with EtBr staining (Sigma-Aldrich, St. Louis, MO, USA) and photographed by Bioimaging system (GELDOC-IT, UVP, Upland, CA, USA). 

***Real-time quantitative reverse transcription PCR (real-time qRT-PCR)*** ***. ***Real-time qRT-PCR was carried out with SYBR Green One-Step qRT-PCR kit (Invitrogen Inc., Carlsbad, CA, USA) under the following conditions: initial reverse transcription at 45°C for 30 min and 95°C for 3 min, followed by 35 cycles of denaturing at 94°C for 45 s, annealing at 55-58°C for 30 s, and extension at 72°C for 45 s. Internal calibration curves were generated by the real-time software (version 2.2). A melting curve analysis was carried out between 60°C and 95°C with a plate read every 0.5°C after holding the temperature for 20 s. The cycle number (*Ct*) at which the signals crossed a threshold set within the logarithmic phase and the peaks of melting curves were recorded. The relative quantitation of gene expression in terms of fold change was calculated using the 2^ΔΔCt^ method [20]. Relative expression levels of target genes in each treatment group were derived from normalizing the Ct value of target genes against that of an endogenous reference (β-actin) and a calibrator (control cells).

***Western blot. ***After washing with cold PBS, the cells were lysed by a lysis buffer, containing 0.01 M Tris, pH 7.5, 0.1 M NaCl, 1% Triton X-100, 0.5% sodium deoxycholate, and 0.1% SDS, with added protease inhibitors. Total proteins in cell lysate were separated by 10% SDS-PAGE and transferred to a polyvinylidene fluoride blotting membrane (Sigma-Aldrich, St. Louis, MO, USA). The membranes were blocked in blocking solution BSA (Sigma-Aldrich, St. Louis, MO, USA) and incubated with mouse monoclonal antibody against Eg5 or KSP (1:200), mouse monoclonal antibody against cyclin D1 (1:500), mouse monoclonal antibody against Bcl-2 (1:500), and mouse monoclonal antibody against Survivin (1:500) (all were bought from Abcam, Cambridge, ENG, UK) at room temperature for 1 h. After washing, the membranes were incubated with horseradish peroxidase-linked goat anti-mouse IgG (1:2000, Abcam, Cambridge, UK) for 45 min. The protein bands were visualized by enhanced chemiluminescence (Sigma-Aldrich, St. Louis, MO, USA). Mouse monoclonal antibody against β-actin (Abcam, Cambridge, UK) was used as a housekeeping gene control. Band intensity was semi-quantitatively analyzed by Image J densitometer (NIH, Bethesda, MD, USA).

***Cell proliferation assay. ***Cell proliferation was measured by WST-1 assay kit (Roche, Basel, Switzer-land). Briefly, siRNA-transfected cells and control cells were seeded at a concentration of 3 × 10^3^ cells per well in 96-well plates (Corning Inc., NY, USA). For indicated time, WST-1 solution was applied at 10 μl per well and incubated at 37°C for 4 h, 5% CO_2_. The absorbance was measured with a microplate ELISA reader (BioTek, Winooski, VT, USA) at 450 nm. The viability was calculated according to the following equation: viability (%) = (OD treated/OD medium) × 100%. The inhibition rate was also calculated according to the following equation: inhibition rate (%) = (1- OD treated/OD control) × 100%.

***Apoptosis assay. ***Apoptosis was investigated by flow cytometry using Annexin V and propidium iodide (BD Biosciences, Franklin Lakes, NJ, USA). Briefly, the cell concentration was firstly adjusted to 1 × 10^6^ cells/ml, and then 1 ml of the cell suspension was taken and centrifuged at 500 ×g at 4^o^C for 10 min. The pellet was rinsed twice with PBS and then re-suspended in a proper volume of binding buffer so that the cell concentration was 5 × 10^4^ cells/ml. After the addition of 10 µl Annexin V-fluorescein isothiocyanate and 5 µl propidium iodide followed by gentle mix, a 15-min reaction was initiated in darkness at room temperature. After that, 300 µl binding buffer was added, and flow cytometry was performed using CellQuest Pro software (BD Biosciences, Franklin Lakes, NJ, USA) to detect cell apoptosis rate (%).

***Anti-tumor drug treatment assay. ***To investigate whether the transfection of KSP-siRNA increases chemosensitivity of Hep3B cells, KSP-siRNA-treated cells were plated at a density of 3 × 10^3^ cells per well in 96-well plates (Corning Inc., NY, USA) and incubated with doxorubicin (Sigma-Aldrich, St. Louis, MO, USA) at various concentrations: 0, 1, 2, and 4 μg/ml for 24 h. Cont-siRNA-treated cells and untreated control cells were also grown under the same conditions. Using WST-1 assay and clonogenic survival assay, cell proliferation was analyzed for indicated time after treatment.

***Clonogenic survival assay. ***The clonogenic survival assay was used to determine the capacity for cell survival and proliferation after radiation or chemotherapy [21]. After treatment with siRNA, cells were seeded at a density of 100 cells per well in 6-well plates for 24 h in a complete medium followed by treatment with different concentrations of doxorubicin: 0, 1, 2, and 4 μg/ml. After 24 h, the medium was replaced with a fresh medium and incubated for additional 10 days. Clones were fixed with methanol and stained with crystal violet (Sigma-Aldrich, St. Louis, MO, USA) for about 15 min. Stained clones that had more than 50 cells were counted at low magnification and cloning efficiency calculated as follows: Cloning efficiency = (Clone number/Total cell number) × 100%.

***Statistical analysis. ***Each experiment was performed in triplicate for all data (n = 3). Data are expressed as mean ± standard error of the mean. Statistical comparisons were performed using the Student’s *t-*test and ANOVA. *P* values < 0.05 were considered to be statistically significant.

## RESULTS

**Fig. 1:**
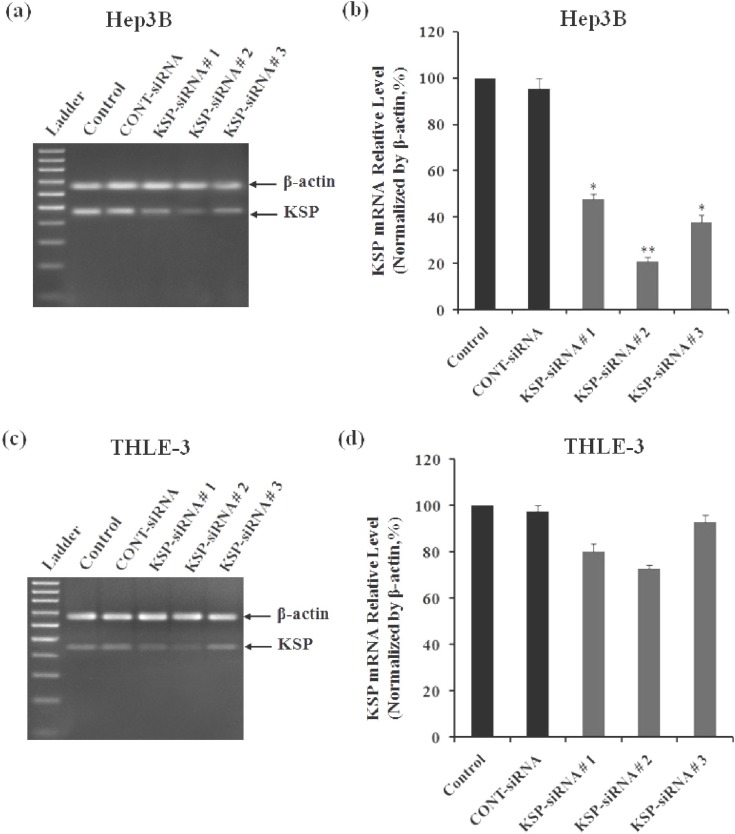
Effect of KSP-siRNA on KSP-mRNA expression in Hep3B cells and THLE-3 cells. Cells were transfected with KSP-siRNA#1, KSP-siRNA#2, KSP-siRNA#3, and Cont-siRNA. Total RNA was extracted from cells at 72 h after siRNA transfection. (a and c) Electrophoretic profiles of PCR products of the KSP (480 bp) and β-actin (680 bp) genes were exhibited in Hep3B cells (a) and THLE-3 cells (c). (b and d) Quantitative analyses of KSP-mRNA level were determined by real-time qRT-PCR after siRNA transfection in Hep3B cells (b) and THLE-3 cells (d). The mRNA expression of KSP was normalized with β-actin. Each bar represents the mean value ± standard deviation (SD) of triplicate. ^*^P<0.05 and ^**^P<0.01 compared to control cell group.

**Fig. 2:**
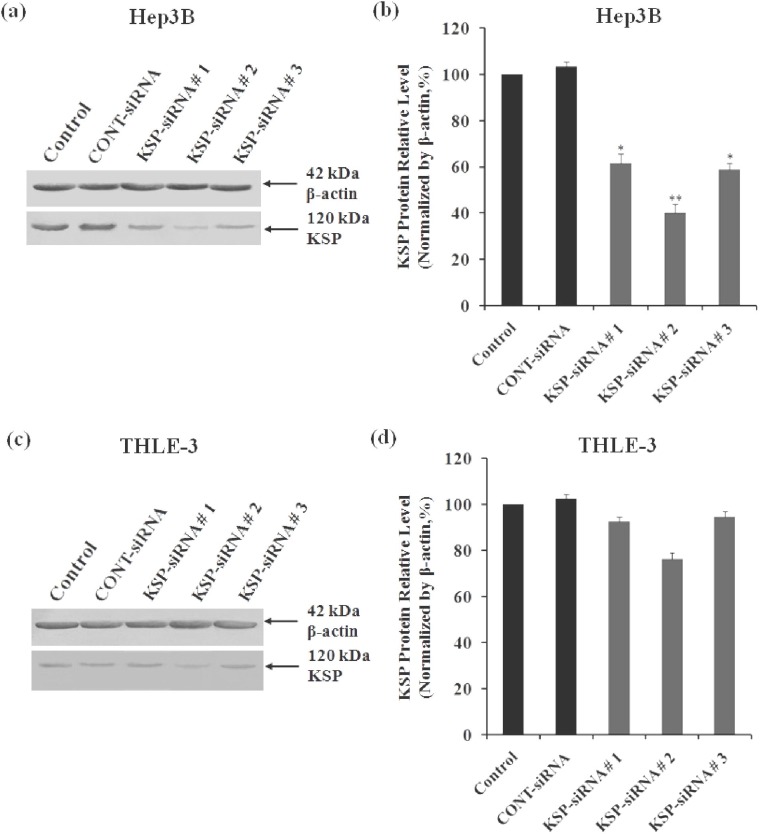
Effect of KSP-siRNAs on KSP protein expression in Hep3B cells and THLE-3 cells. Total cellular protein was extracted from cells at 72h after siRNA transfection. After electrophoresis and electrotransfer to membranes, the membranes were incubated with mouse Antibody against KSP and then with horseradish peroxidase-linked goat anti-mouse IgG. (a and c) The expressions of KSP were examined by Western blot analyses in Hep3B cells (a) and THLE-3 cells (c). β-actin was used as a housekeeping gene control. The size of each protein was indicated. (b and d) The KSP-siRNA-transfected cells exhibited a decreased expression of KSP protein as confirmed by densitometric analysis in Hep3B cells (b) and THLE-3 cells (d). Each bar represents the mean value ± standard deviation (SD) of triplicate. ^*^P<0.05 and ^**^P<0.01 compared to control cell group.

***The expression of endogenous KSP in Hep3B cells and THLE-3 cells. ***In this study, the expression of endogenous KSP at mRNA and protein levels were determined in both HCC cell line Hep3B, and normal liver cell line THLE-3 by RT-PCR and Western-blot analyses. Density of KSP band in RT-PCR analyses showed that endogenous KSP expression at mRNA level was higher in Hep3B cells compared to THLE-3 cells (control cells in Fig. 1a and c). It was also confirmed at protein level by Western-blot analyses (control cells in Fig. 2a and c).

***Effects of KSP-siRNA on KSP expression in Hep3B cells and THLE-3 cells. ***To address the functions of KSP, both Hep3B cells and THLE-3 cells were transfected with KSP-siRNA and Cont-siRNA. Subsequently, the efficiency of KSP silencing was determined by analysis of the levels of KSP-mRNA and protein. For validation purposes, three different siRNA (KSP-siRNA#1-3) targeting different regions of human KSP were employed (Table 1). Then, one siRNA with best repressive effect was used in following experiments. 

**Fig. 3:**
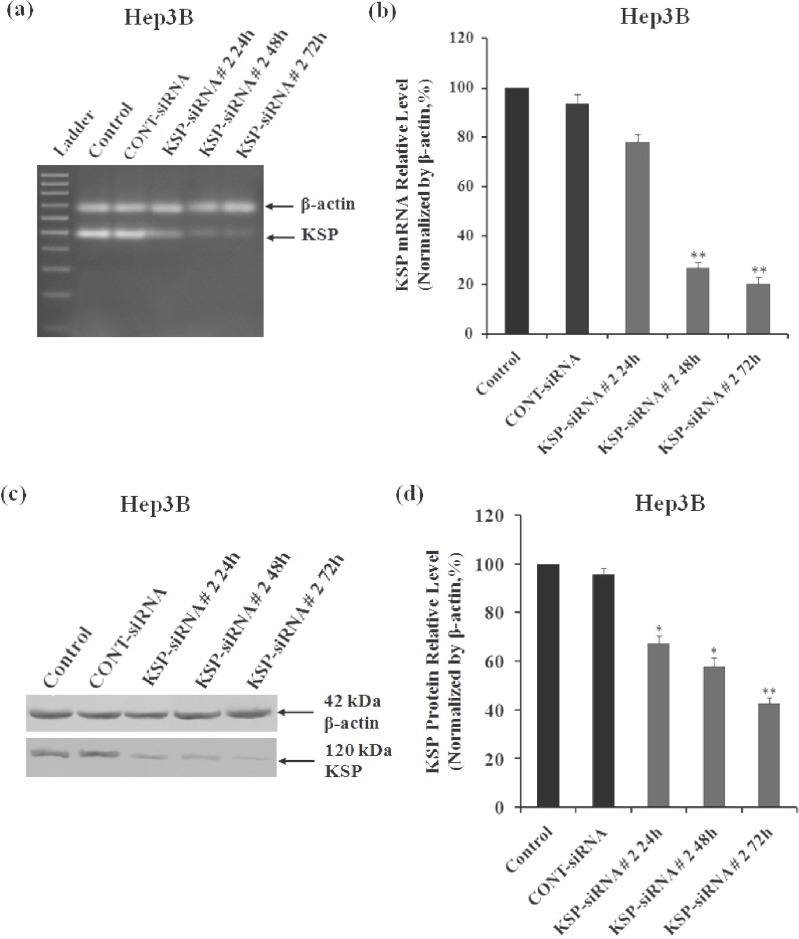
Effect of KSP-siRNA#2 on KSP expression in Hep3B cells. Cells were transfected with KSP-siRNA# 2 and Cont-siRNA and then harvested at indicated times. (a and b) Total RNA was extracted from cells at 0 h, 24 h, 48 h and 72 h after KSP-siRNA#2 transfection. The expressions of mRNA KSP were examined by RT-PCR (a) and real-time qRT-PCR (b) after siRNA transfection. (c and d). Total cellular protein was extracted from cells at 0 h, 24 h, 48 h and 72 h after siRNA transfection. The expressions of protein KSP were examined by Western blot analyses (c) and densitometric analyses (d). β-actin was used as a housekeeping gene control. Each bar represents the mean value ± standard deviation (SD) of triplicate. ^*^P<0.05 and ^**^P<0.01 compared to control cell group.

In THLE-3 cells, KSP-siRNA had less influence on KSP expression at both mRNA and protein levels. However, there was no significant difference in the expression of KSP between KSP-siRNA-treated cells or Cont-siRNA-treated cells and untreated control ones (Fig. 1c and d, Fig. 2c and d). In contrast, in examined Hep3B cells, all three KSP-siRNA had noticeable effects on KSP expression. Among them, KSP-siRNA#2 was the most effective in reducing KSP-mRNA, and the downregulation was correlated with a remarkable decrease in target protein (Fig. 1a and b, Fig. 2a and b). Therefore, KSP-siRNA#2 was selected to evaluate its effects on KSP expression in Hep3B cells at different time intervals after transfection. The results of RT-PCR experiments on total RNA, obtained from the KSP-siRNA#2-Hep3B cells, indicated downregulation of KSP-mRNA in comparison to that of control cells and Cont-siRNA-treated cells after 72 h. The density of KSP band showed that KSP-mRNA expression was blocked clearly after 48 h, and inhibition was stabled up to 72 h after transfection (Fig. 3a). In addition, the relative amounts of KSP-mRNA in each sample were quantified using real-time qRT-PCR. The results of real-time qRT-PCR analyses indicated that mRNA expression level of KSP in KSP-siRNA#2-transfected cells began to alter within 24 h after transfection, from 100% to 77.85 ± 3.15%. The mRNA expression level was strongly decreased after 48 h (26.82 ± 2.07%) and 72 h (20.47 ± 2.69%) as compared to that of the control cells (*p* < 0.01), while it was not much altered in Cont-siRNA-transfected cells during 72 h after transfection (93.35 ± 3.85%) (Fig. 3b). These values indicated that KSP-siRNA#2 triggered a 79.53 ± 2.69% decrease in the KSP-mRNA expression, whereas Cont-siRNA-mediated mRNA downregulation was about 6.65 ± 3.85% at 72 h. The regulatory effects of the KSP-siRNA#2 on KSP protein expression in Hep3B cells were determined by Western-blot. The results showed that KSP-siRNA#2-transfected cells expressed significantly less KSP protein than control cells or Cont-siRNA-treated cells after 72 h (Fig. 3c). The densitometric analyses also confirmed that KSP expression in post-transfected cells was effectively inhibited by KSP-siRNA#2 at protein levels by 32.52 ± 2.82% after 24 h, and the inhibition was stabled up to 72 h (the protein level by 57.25 ± 2.47%) compared to control cells (*P*<0.01) (Fig. 3d). These data showed that KSP-silencing efficiency by KSP-siRNA might correlate with the extent of endogenous KSP expression which was higher in cancerous cell line Hep3B compared to non-cancerous cell line THLE-3. 

**Fig. 4:**
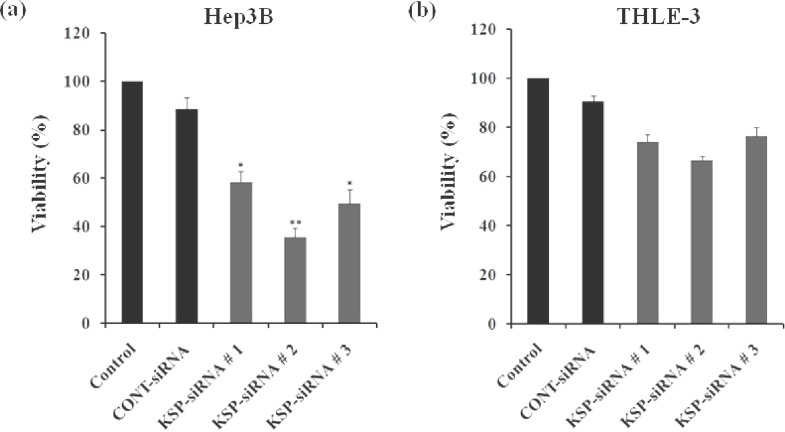
Effect of KSP-siRNA on the cell viability. The viability of Hep3B cells and THLE-3 cells treated with siRNA at 72h were measured using WST-1 assay. The cell viability was expressed as the percentage of control cells. The viability of Hep3B cells (a) and the viability of THLE-3 cells (b) were shown for each group. Data represent the mean value ± SD of triplicate. ^*^P<0.05 and ^**^P<0.01 compared to control cell group.

***Effects of KSP-siRNA on cell proliferation in Hep3B cells and THLE-3 cells. ***The effects of siRNA on the viability of both Hep3B cells and THLE-3 cells were determined at 72 h after transfection (Fig. 4). The cell viability was examined by WST-1 cell pro-liferation assays. In Hep3B cells, all three KSP-siRNA induced a significant reduction in cell viability compared to untreated control cells (*P*<0.05) (Fig. 4a). The results clearly showed that cell proliferation was markedly inhibited by KSP-siRNA. Consistent with the changes in KSP expression, KSP-siRNA#2 also caused the greatest suppression on cell growth, thus selecting for further studies. Compared to Hep3B cells, KSP-siRNA displayed no or lower effects on the viability of THLE-3 cells (Fig. 4b). In addition, we did not observe any significant phenotypic differences when KSP-siRNA was used in THLE-3 cells (data not shown). In both Hep3B cells and THLE-3 cells, transfection with Cont-siRNA did not affect cell viability. On the other hand, the negative Cont-siRNA had relatively limited off-target effects on cell proliferation as expected. These results displayed that cell growth inhibition was conformable with the extent of KSP silencing by siRNA in both Hep3B cells and THLE-3 cells.

**Fig. 5:**
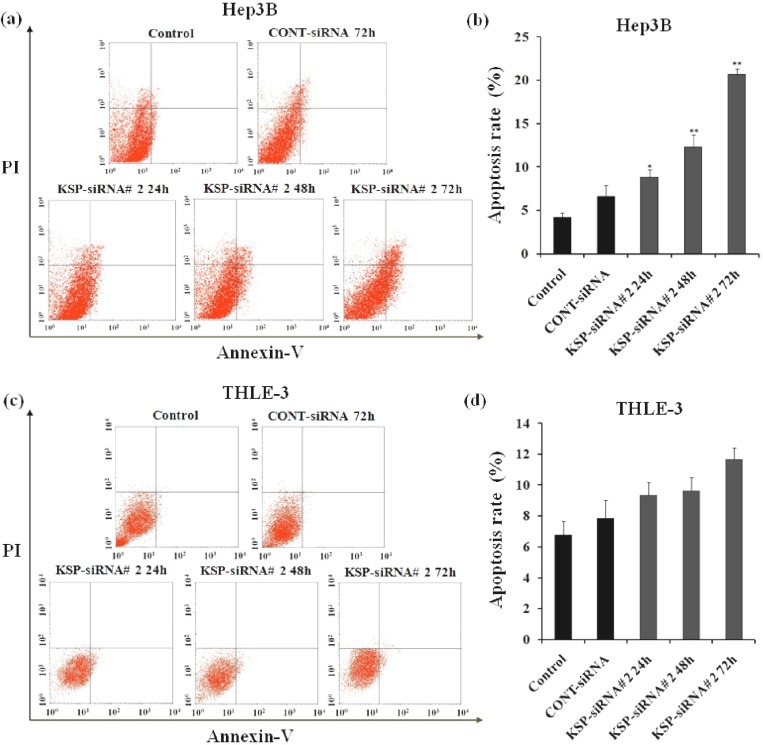
Effect of KSP-siRNA#2 on the induction of apoptosis in Hep3B cells and THLE-3 cells. (a and c) Cell apoptosis was detected by flow cytometry in Hep3B cells (a) and THLE-3 cells (c). Cells in the lower left (LL) quadrant represented survivals; lower right (LR) quadrant represented early apoptosis; the upper right (UR) quadrant represented necrosis or post-apoptotic and the upper left (UL) quadrant represented detection of error allowed. (b and d) Data show the mean ± SD intensity of fluorescent positive cells during early apoptotic events of triplicate in Hep3B cells (b) and THLE-3 cells (d). ^*^P<0.05 and ^**^P< 0.01 compared to control cell group.

***Effects of KSP-siRNA#2 on apoptosis*** ***in Hep3B cells and THLE-3 cells. ***The growth inhibition in KSP-downregulated Hep3B cells could be attributed in part to cell apoptosis. The apoptosis was assessed by Annexin-V staining methods. In the staining method, the percentage of apoptotic cells was scored by using flow cytometry. Our results revealed induction apoptosis in Hep3B cells after treatment with KSP-siRNA#2 (Fig. 5a). The percentage of apoptotic cells by KSP-siRNA#2 treatment was exponentially changed after 24 h (8.86 ± 0.78%) and significantly increased after 48 h (12.25 ± 1.42%) and 72 h (20.64 ± 0.65%), respectively as compared to that of control cells (4.15 ± 0.66%) (*P*<0.01), whereas the apoptosis rate of Cont-siRNA-treated cells at 72 h was approximately 6.62 ± 1.25% (Fig. 5b). Consequently, the apoptosis rate of the Hep3B cells transfected with KSP-siRNA#2 was higher than control cells or Cont-siRNA-treated cells. However, the result was not reproduced in THLE-3 cells, which showed no significant difference in apoptosis rate between KSP-siRNA#2 or Cont-siRNA-treated cells and control cells as a consequence of KSP silencing (Fig. 5c and d). It was demonstrated that siRNA-mediated down-regulation of KSP in Hep3B cells could induce apoptosis, leading to inhibition of proliferation.

**Fig. 6:**
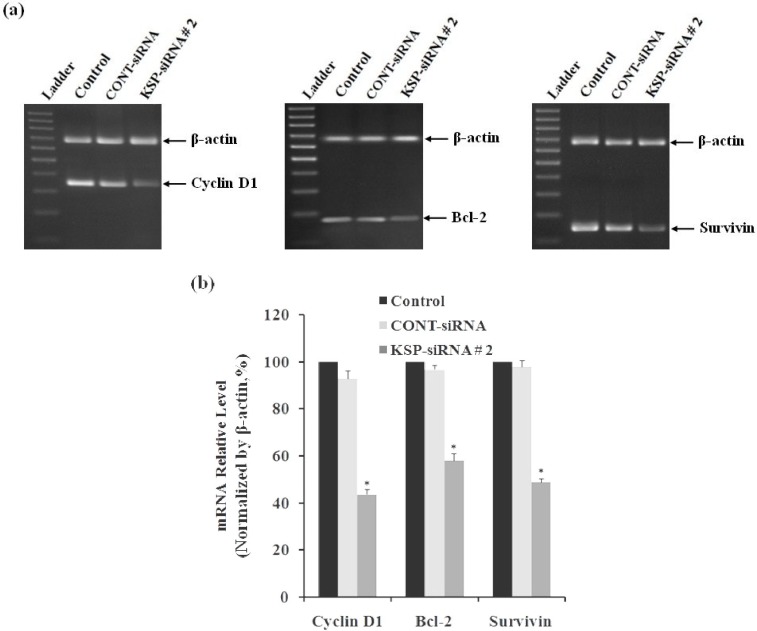
The mRNA expression of Cyclin D1, Bcl-2 and Survivin in Hep3B cells. (a) The mRNA expressions of Cyclin D1, Bcl-2 and Survivin in Hep3B cells were detected by RT-PCR after 72 h of siRNA transfection. Electrophoretic profile of PCR products of the Cyclin D1 (358 bp) Bcl-2 (174 bp), Survivin (145 bp) and β-actin (680 bp) genes. β-actin was used as a housekeeping gene control. (b) The mRNA levels of Cyclin D1, Bcl-2 and Survivin in Hep3B cells were also determined by real-time qRT-PCR. The mRNA expression of these genes was normalized with β-actin. Each bar represents the mean value ± standard deviation (SD) of triplicate. ^*^P<0.05 compared to untreated cell group.

**Fig. 7:**
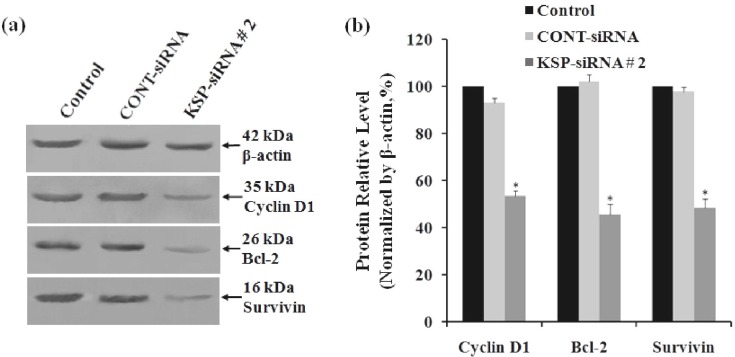
The protein expression of Cyclin D1, Bcl-2 and Survivin in Hep3B cells. (a) The protein expressions of Cyclin D1, Bcl-2 and Survivin in Hep3B cells were measured by Western blot analyses after transfection. β-actin was used as a housekeeping gene control. The size of each protein was indicated. (b) Densitometric analysis of these three proteins was made relative to β-actin. Each bar represents the mean value ± standard deviation (SD) of triplicate. ^*^P<0.05 compared to control cell group

***The molecular mechanisms underlying the growth inhibitory effects and apoptosis induction of KSP*** ***downregulation*** ***in Hep3B cells. ***To identify the molecular targets and common mechanisms underlying the growth inhibitory effects and apoptosis induction caused by KSP silencing, Hep3B cells were treated with either Cont-siRNA or KSP-siRNA#2 for 72 h and subjected to analysis anti-apoptosis gene expression, including *cyclin D1*, *Bcl*-*2, and survivin*. The results indicated that downstream targets of *cyclin D1*, *Bcl*-*2, and survivin* were also downregulated at *both* protein and mRNA levels. The band intensity of RT-PCR products showed the expression levels of *cyclin D1*, *Bcl*-*2, and survivin* mRNA were lower than those of control cells and Cont-siRNA-treated cells, after 72 h (Fig. 6a). The relative levels of mRNA of *cyclin D1*, *Bcl*-*2, and survivin* were also determined using real-time RT-qPCR after 72 h of siRNA transfection. The mRNA levels of cyclin D1 and Bcl-2 were downregulated by 56.35 ± 2.25% and 43.12 ± 3.02%, respectively, whereas the mRNA levels of *survivin* were downregulated by 51.34 ± 1.58% in KSP-siRNA#2-transfected cells compared to those in control cells (*P*<0.05) (Fig. 6b). Similarly, the expression levels of cyclin D1, Bcl-2, and survivin proteins were measured using Western-blot analyses after trans-fection, indicating similar results (Fig. 7a). KSP-siRNA#2 inhibited cyclin D1, Bcl-2, and survivin expression at the protein level up to 46.55 ± 2.26%, 54.35 ± 4.32%, and 51.56 ± 3.78%, respectively in comparison to control cells (*P*<0.05) (Fig. 7b). However, there was no significant difference in mRNA and protein levels of cyclin D1, Bcl-2, and survivin between Cont-siRNA-treated cells and untreated control cells.

**Fig. 8:**
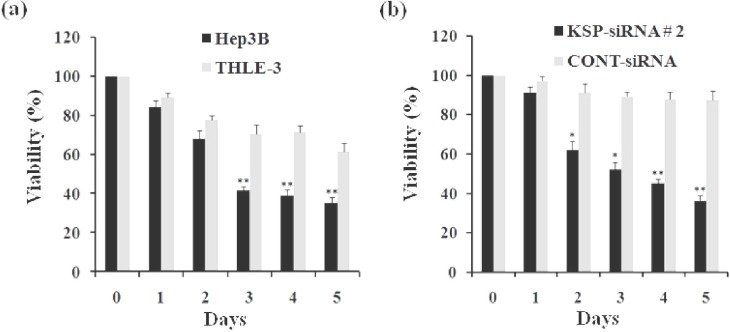
Effect of KSP-siRNA#2 treatment on the cell viability. (a) The viability of Hep3B cells and THLE-3 cells treated with KSP-siRNA#2 for indicated time in WST-1 assay, cell viability was expressed as the percentage of control cells (0 day). (b) The viability of Hep3B cells treated with the same concentration of KSP-siRNA#2 and Cont-siRNA for indicated time in WST-1 assay, cell viability was then quantified as described above. All results shown were means ±SD of triplicate. ^*^P<0.05 and ^**^P<0.01 compared to control cell group.

***Inhibition of*** ***KSP downregulated Hep3B*** ***cell proliferation after*** ***treatment*** ***with*** ***doxorubicin. ***To evaluate the effects of KSP-siRNA#2 on the viability of Hep3B cells or THLE-3 cells, cells were treated for five days. The viability was determined using WST-1 assay. The results showed that KSP-siRNA#2 significantly decreased the viability of Hep3B cells in a time-dependent manner. However, KSP-siRNA#2 had less effect on the viability of THLE-3 cells (Fig. 8a). These findings indicated that Hep3B cells, but not THLE-3 cells, were sensitive to KSP-siRNA. In addition, we also compared the cytotoxicity of Cont-siRNA and KSP-siRNA#2 toward Hep3B cells for five days. Cells were treated with Cont-siRNA or KSP-siRNA#2 at same concentration. The results showed that KSP-siRNA#2, but not Cont-siRNA, can directly mediate cytotoxicity toward Hep3B cells (Fig. 8b).

**Fig. 9:**
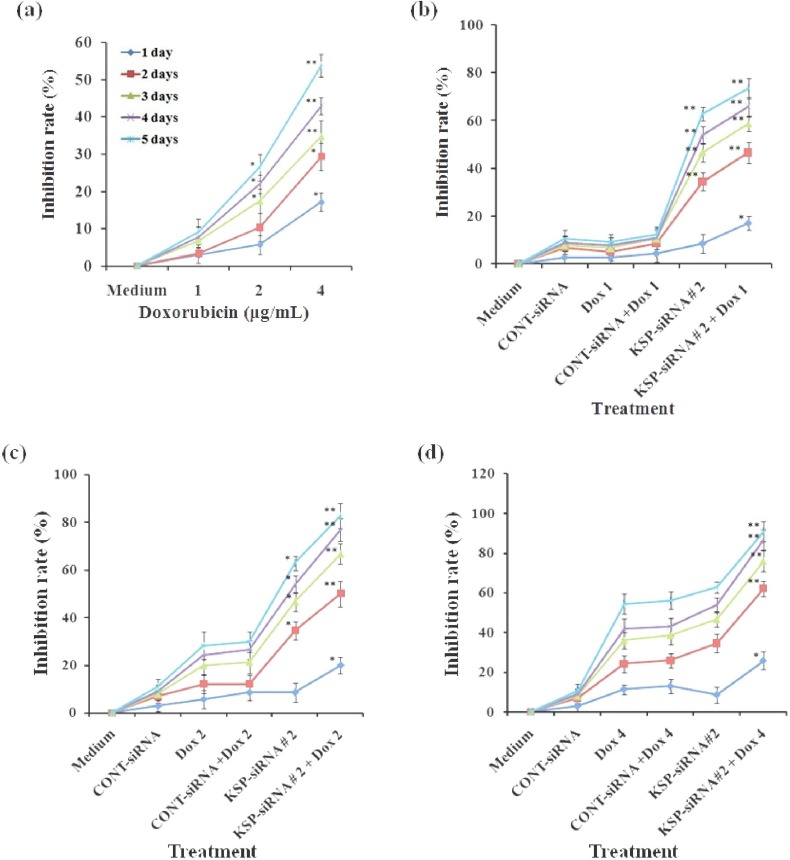
Effect of KSP-siRNA#2 and/or doxorubicin treatment on the inhibition of cell proliferation. (a) Hep3B cells were treated with indicated concentration of doxorubicin for indicated time in WST-1 assay. The results were presented as the inhibitory ratio of doxorubicin treated cells. (b, c, and d) Hep3B cells treated with KSP-siRNA#2, Cont-siRNA, doxorubicin and KSP-siRNA#2 or Cont-siRNA in combination with doxorubicin for indicated time, inhibition was then determined by the WST-1 assay. All results shown were means ± SD of triplicate. ^*^P<0.05 and ^**^P<0.01 compared to medium (a) or doxorubicin alone treated cell group (b, c, and d).

**Fig. 10:**
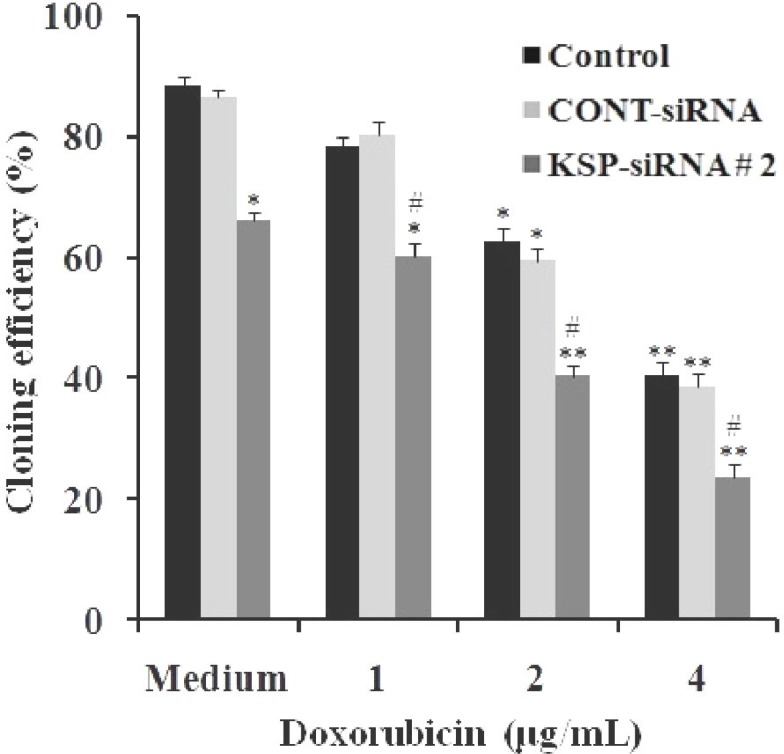
Comparison of cloning efficiency at different doxorubicin concentrations. At the same concentration of drug, cloning efficiency declined notably in KSP-siRNA#2 treated cell group, but not in Cont-siRNA treated cell group compared to the doxorubicin treated cell group. Each bar represents the mean value ± standard deviation (SD) of triplicate. ^*^P<0.05 and ^**^P<0.01 compared to control cell group, ^#^*p* < 0.05 compared to control cell group treated at the same concentration of doxorubicin.

To evaluate the inhibition effect of doxorubicin treatment on Hep3B cells, cells following treated with doxorubicin at designated concentrations for the indicated time were carried out in WST-1 assay and clonogenic assay. It was clear that doxorubicin alone could significantly reduce Hep3B cell growth, and the inhibitory effect exhibited in a dose- and time-dependent manner (Fig. 9a). However, the effects of doxorubicin on Hep3B cells were not observable at concentration of 1 μg/ml. At 4 μg/ml, the inhibition by doxorubicin on cell proliferation became apparent, with the inhibition rate value increasing from 17.14 ± 2.46% at day one to 51.71 ± 3.03% at day five. In addition, cloning efficiency was declined significantly in cells following treatment of 4 μg/ml doxorubicin dose when compared to control cells (*P*<0.01) (Fig. 10).

**Fig. 11:**
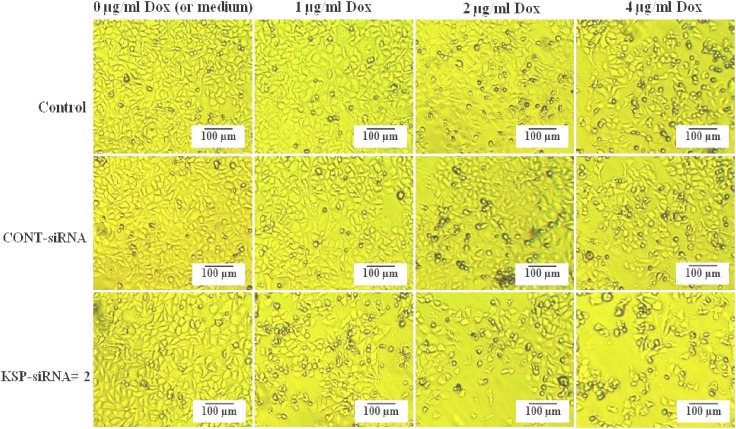
The cells after treatment at different doxorubicin concentration. Control cells, Cont-siRNA treated cells and KSP-siRNA#2 treated cells were treated with indicated concentrations of doxorubicin: 0 (or medium), 1, 2, and 4 μg/mL. Pictures were captured at day 3.

In order to evaluate the synergistic effect of KSP-siRNA#2 and doxorubicin on Hep3B cells, cells following treated with KSP-siRNA#2 or Cont-siRNA in presence or absence of doxorubicin were carried out in WST-1 assay and clonogenic survival assay. The results indicated that doxorubicin effects were noticeable in the KSP downregulated cells. As illustrated in Figure 9b, after five-day treatment, KSP-siRNA#2 in combination to 1 μg/ml doxorubicin could increase inhibition rate (71.55 ± 4.36%) when compared to KSP-siRNA#2 alone (58.03 ± 2.87%) or doxorubicin alone (9.09 ± 3.54%) (*P*<0.01). However, there was no significant difference in inhibition of cell growth between Cont-siRNA plus 1 μg/ml doxorubicin or Cont-siRNA and doxorubicin alone. To further determine if KSP-siRNA#2 can enhance the chemosensitivity of doxorubicin-treated Hep3B cells, KSP-siRNA#2-treated cells as well as Cont-siRNA-treated cells and control cells were treated with higher doses of doxorubicin (2 and 4 μg/ml) for five days. For KSP-siRNA#2 plus 2 μg/ml or 4 μg/ml doxorubicin groups, the inhibition rates were 80.64 ± 5.23% and 0.91 ± 5.07%, respectively. For Cont-siRNA plus 2 μg/ml or 4 μg/ml doxorubicin groups, the inhibition 9 rates were 28.85 ± 4.30% and 55.20 ± 4.16%, respectively. For 2 μg/ml or 4 μg/ml doxorubicin alone groups, the inhibition rates were 26.38 ± 4.87% and 54.46 ± 5.03%, respectively (Fig. 9c and d). In addition, the KSP-downregulated cells showed no sign of proliferation, with necrosis observed at day three after doxorubicin treatment (Fig. 11). Obviously, treatment with a series of doxorubicin doses in the presence of KSP-siRNA#2 increased the cell inhibition compared to treatment with doxorubicin and/or Cont-siRNA, further supporting the synergistic effect. In other words, KSP-siRNA transfer can increase the doxorubicin chemosensitivity of Hep3B cell. It is also noted that the synergistic cytotoxic effect is effective, even at low dose (1 μg/ml) compared to control. These results were also further supported by clonogenic survival assay. A significant decline in cloning efficiency was observed in the combination of KSP-siRNA#2 and doxorubicin as compared to KSP-siRNA#2 or doxorubicin alone (Fig. 10).

## DISCUSSION

Several aspects of KSP or Eg5 biology make it an excellent indicator for monitoring RNAi. First, the endogenous KSP gene is expressed in all the proliferating cells that have been analyzed to date. Second, its activity is required for mitosis, and therefore the penetration of siRNA systematically results in an inhibition of growth. This growth inhibition can be evaluated by several approaches, from cell counting to DAPI staining and either microscopy or cytofluorometry. Finally, the action of KSP-siRNA is rapid and best analyzed between 30 h and 48 h. The evaluation of siRNA transfection in cells can therefore be easily monitored with no specific reagents required [16]. Therefore, inducing a degradation of KSP by siRNA was expected to lead to a novel approach for the control of cancer cells. To decrease expression of KSP, we used RNAi technology to transfect siRNA targeting KSP into cancerous cell line Hep3B and non-cancerous cell line THLE-3, as a control. Several siRNA targeting against different regions of human KSP were used routinely to ensure and possibly enhance silencing. Following KSP-siRNA transfection, cells were subjected to RT-PCR, real-time qRT-PCR, Western-blot, and drug effects were investigated. Transfection of KSP-siRNA into Hep3B cells reduced the expression of KSP at both mRNA and protein levels. RT-PCR and real-time qRT-PCR analyses showed that KSP-mRNA remaining in the KSP-siRNA-treated cells was much lower than control cells and Cont-siRNA-treated cells. At translational level, KSP protein level of post-transfected Hep3B cells were assessed by Western-blot, confirming a noticeable reduction in KSP expression. Our observations are in consistent with the previous reports that used KSP-siRNA to monitor KSP expression in different cancer cells, including colon carcinoma cells, lung carcinoma cells, breast carcinoma cells, prostatic adenocarcinoma cells, and ovary cancer cells [6, 13, 22]. In contrast, in normal THLE-3 cells, the expression of KSP in KSP-siRNA-transfected cells was unaltered when compared to control cells. KSP expression is elevated in tumor samples compared with adjacent normal tissues in breast, colon, liver, lung, ovary, rectal, and uterus, consistent with its role in proliferating cells [23]. Consequently, the low levels of endogenous KSP expression may be a reason for decrease of KSP-silencing. This data is also partially supported by a previous study which revealed a correlation between the silencing efficiency of siRNA and the intracellular transcript levels of target genes on different cell lines. The low abundant transcripts are less susceptible to siRNA-mediated degradation than the medium or high abundant transcripts [24]. Furthermore, gene silencing efficiency of siRNA may depend on specific features of the RNAi machinery in each cell lines [22] and the local structure of mRNA at the targeted region [25].

Inhibition of KSP activity, either by microinjection of antibodies [26] or with a specific drug such as monoastrol [15], leads to a monopolar spindle and an arrest of cells in prometaphase and ultimately to cell death. In this work, we also demonstrated that KSP- siRNA#2 could inhibit cell proliferation and induce apoptosis in cancerous Hep3B cells, but not in non-cancerous THLE-3 cells. These results are in agreement with the previous observations when using several siRNA targeting KSP on both normal cells and cancer cells [13, 22]. The effects of KSP knockdown are also known to vary depending on cell line and can either inhibit cell proliferation or induce apoptosis [27].

To elucidate the molecular mechanisms by which KSP-siRNA inhibit proliferation and induce apoptosis of Hep3B cells, we have examined the expressions of the key regulators cyclin D1, Bcl-2, and survivin. Our results first demonstrated that the expression levels of cyclin D1, Bcl-2, and survivin were significantly decreased in Hep3B cells after transfection with of KSP-siRNA. Cyclin D1 is known to accumulate during the G1 phase of the cell cycle [28]. Overexpression of cyclin D1 may be an early event in hepato-carcinogenesis, and it plays a role in tumor growth and differentiation [29, 30]. In contrast, Bcl-2 and survivin are thought to be very important anti-apoptotic proteins in cells. They are identified to be one of the mechanisms involved by cancer cells to evade apoptosis. Bcl-2 is the prominent member of a protein family that is responsible for dysregulation of apoptosis and prevention of death in cancer cells, which controls the pathways leading to the release of cytochrome c from the mitochondrial membrane, the activation of caspase cascade and, in the end, to execution of apoptosis [31]. Overexpression of Bcl-2 may protect human hepatoma cells from antibody-mediated apoptosis [32]. Similarly, survivin is a new member of inhibitors of the apoptosis protein family that have been implicated in both cell division and inhibition of apoptosis. By inhibiting apoptosis and promoting mitosis, survivin facilitates cancer cell survival and growth. The overexpression of survivin in liver cancer can protect cells from apoptosis and promote cell cycle progression by inhibiting pro-apoptotic caspases-3 and caspases-7 [33, 34]. From our findings, we surmised that the downregulation of cyclin D1, Bcl-2, and survivin expressions by siRNA-KSP transfection was one of the important ways of inducing cell apoptosis, subsequently leading cell death.

Drug resistance is a major problem in cancer treatment with chemotherapy, since after long term exposure to chemotherapeutic agent, cancer cells may no longer respond to the treatment. Or more too often, cancer cells, due to the intrinsic instability of their genome, may develop resistance to several completely different chemotherapeutic agents simultaneously, also known as multidrug resistance [35]. Thus, the approach of enhancing chemosensitivity by using apoptosis-inducing agents appears to be a potential approach for more efficient cancer treatment. The inhibition of KSP expression by siRNA is anticipated to an increase in the chemosensitivity of cancer cells with anti-tumor drugs. Among anti-tumor drugs, doxorubicin is a commonly used anti-cancer drug causing DNA damage and killing cancer cells mainly by apoptosis. Doxo-rubicin is also used in many researches, especially on anti-tumor drug resistance. However, the process leading to death of cancer cells and molecular basis of resistance to doxorubicin are not well understood. In our study, Hep3B cells were treated with a series of doses of doxorubicin. The effects of doxorubicin on normal cells were not observable at low concentration. At higher doses, the effects on proliferation inhibition became apparent. It was suggested that Hep3B cells has strong resistance to doxorubicin. The resistance to doxorubicin was decreased by downregulation of KSP. In other words, the KSP knockdown cells were more sensitive significantly to doxorubicin compared to the original cells. Even at high doxorubicin concentrations, the drug failed to kill normal cancer cells, whereas it caused death in the KSP-siRNA-transfected cells at lower concentrations. This implies that the reduction of KSP expression mitigated the drug-resistant ability of HCC cells. These findings also further supported a correlation between drug resistance and KSP expression or signal pathways related to this protein [14, 36]. Thus, the combination of siRNA and anti-tumor may be an efficient therapy for liver cancer treatment, which was also confirmed in recent studies [37-39]. Based on the results of this study, KSP is obviously the target of intense research for development of novel anti-cancer therapeutics. 

Blocking KSP expression using siRNA significantly reduces KSP-mRNA and protein levels in Hep3B cells. KSP-siRNA suppresses cell proliferation and induces apoptosis through the downregulation of cyclin D1, Bcl-2, and survivin. Our data also indicated that the decrease of KSP expression made HCC cells more sensitive to anti-cancer drugs. A combination of gene therapy to reduce KSP expression and chemotherapy alleviated drug resistance in treated cells. This method could be a new targeted strategy to eradicate HCC cells *in vitro*.
